# Eating at Night

**Published:** 1898-10

**Authors:** 


### Eating at Night.

A writer in the *Italia Termale* declares in favor of late suppers. He says that many people who remain thin and weakly in spite of all precautions in regard to diet, etc., owe the fact to habitual abstemiousness at night. He remarks that physiology teaches us that in sleeping as in waking there is a perpetual waste going on in the tissues of the body, and it seems but logical that nourishment should be continuous as well. The digestion of the food taken at dinner or in the early evening is finished as a usual thing before or by bedtime, yet the activity of the process of assimilation, etc., continues for hours afterward; and when one retires with an empty stomach the result of this activity is sleeplessness and an undue wasting of the system. All other creatures, says the writer, outside of man, are governed by a natural instinct which leads those having a stomach to eat before lying down for the night. The infant, guided by the same instinct, takes the breast frequently in the night as well as in the day, and if its stomach is allowed to remain empty too long it shows its discomfort by crying. The digestive organs have no need for repose, provided always that the quantity of nourishment taken within the twenty-four hours does not go beyond the normal limit. The fact that the intervals between meals is short, works no inconvenience, but on the contrary tends to the avoidance of feebleness which is the natural result of an interval extended to too great a length. Feeble persons, lean and emaciated persons, and above all those suffering from insomnia, owe it to themselves not to retire without taking some nourishment into the stomach—bread and butter, a glass of rich milk, a few biscuits, or even a bit of juicy, cold meat for instance.—*Medical Record.*

---

**The New York Quarantine Station** has imposed upon it a stupendous task, which it barely meets, owing to the arrival of the various army hospital vessels from southern latitudes where yellow fever is a constant menace. The *New York Tribune* gives a cut of the disinfecting boat, the James W. Wadsworth, on duty alongside a newly arrived steamer. Dr. Alvah H. Doty, the health officer of the port, has long since discarded alleged disinfections by sulphur and substituted steam in lieu thereof, a method first suggested by Dr. Edward R. Squibb, of Brooklyn, New York. At first there were many obstacles to be overcome, but finally the present apparatus, in use for two years, fulfilled all expectations. The Harvard, the largest vessel disinfected, underwent the process in two days' time, and thus saved to the government hundreds of dollars a day in the speedy release to her duties. Under the old process two or perhaps three weeks would have been consumed. The vessel in question, it must be remembered, according to Dr. Doty's account, had about 500 men aboard, every one of whom had clothing and bundles by no means above suspicion. The steam used had a temperature of 230 degrees F. or over, but formaldehyde gas is also resorted to in instances where injury by steam to fabrics, such as silk, laces, leather goods, etc., would result. Other devices for the prevention of communication by the development of incubative cases are used.—*Journal of the American Medical Association.*

